# Elucidating the genomic history of commercially used *Bacillus thuringiensis* subsp. *tenebrionis* strain NB176

**DOI:** 10.3389/fcimb.2023.1129177

**Published:** 2023-03-20

**Authors:** Lea Schäfer, Frank Volk, Regina G. Kleespies, Johannes A. Jehle, Jörg T. Wennmann

**Affiliations:** ^1^ Julius Kühn Institute (JKI) - Federal Research Centre for Cultivated Plants, Institute for Biological Control, Dossenheim, Germany; ^2^ Biofa GmbH, Münsingen, Germany

**Keywords:** *Bacillus thuringiensis* subsp. *tenebrionis*, *Bacillus cereus* group, whole genome sequencing, nanopore, enterotoxin, *cry3Aa*

## Abstract

*Bacillus thuringiensis* subsp. *tenebrionis* (Btt) produces a coleopteran-specific crystal protoxin protein (Cry3Aa δ-endotoxin). After its discovery in 1982, the strain NB125 (DSM 5526) was eventually registered in 1990 to control the Colorado potato beetle (*Leptinotarsa decemlineata*). Gamma-irradiation of NB125 resulted in strain NB176-1 (DSM 5480) that exhibited higher cry3Aa production and became the active ingredient of the plant protection product Novodor^®^ FC. Here, we report a comparative genome analysis of the parental strain NB125, its derivative NB176-1 and the current commercial production strain NB176. The entire genome sequences of the parental and derivative strains were deciphered by a hybrid *de novo* approach using short (Illumina) and long (Nanopore) read sequencing techniques. Genome assembly revealed a chromosome of 5.4 to 5.6 Mbp and six plasmids with a size range from 14.9 to 250.5 kbp for each strain. The major differences among the original NB125 and the derivative strains NB176-1 and NB176 were an additional copy of the *cry3Aa* gene, which translocated to another plasmid as well as a chromosomal deletion (~ 178 kbp) in NB176. The assembled genome sequences were further analyzed *in silico* for the presence of virulence and antimicrobial resistance (AMR) genes.

## 1 Introduction

*Bacillus thuringiensis* (Bt) is a rod-shaped, spore-forming, gram-positive, and entomopathogenic bacterium (Ibrahim et al., 2010; Jouzani et al., 2017). During sporulation, Bt produces parasporal crystals, consisting of one or more insect-specific crystal (Cry) and/or cytolytic (Cyt) proteinaceous protoxins (δ-endotoxins) (Bravo et al., 2011; Crickmore et al., 2021). After oral ingestion by insect larvae, these proteins are proteolytically activated in the midgut and bind to specific receptors located in the midgut epithelial cell membrane, leading to intestinal perforation, feeding stop and finally insect death (Schnepf et al., 1998; Bravo et al., 2007). The δ-endotoxins are encoded by *cry* and/or *cyt* genes, typically located on large plasmids (Carlton, 1980; Kronstad et al., 1983; Carlson et al., 1996). With the discovery of Bt strains specific to Lepidoptera, Diptera or Coleoptera, many Bt strains have been successfully developed and used as highly specific biological control agents (Bravo et al., 2007).

The coleopteran specific *Bacillus thuringiensis* subsp. *tenebrionis* (Btt) (strain BI 256-82) was first isolated from an infected pupa of the yellow mealworm *Tenebrio molitor* L. (Coleoptera: Tenebrionidae) at the Institute for Biological Control in Darmstadt, Germany, in 1982 (Krieg et al., 1983). According to serological investigations, the strain was assigned to serovar “morrisoni” (H8a8b) (Krieg et al., 1987). The coleopteran activity of this strain resides in a flat, plate-like crystal, composed primarily of CrylllA (now Cry3Aa) (Höfte et al., 1987; Höfte and Whiteley, 1989; Crickmore et al., 1998; Crickmore et al., 2021). The original isolate of Btt, strain BI 256-82, was patented (US 4,766,203 and US 4,889,918) and deposited at the Leibniz Institute DSMZ-German Collection of Microorganisms and Cell Cultures GmbH (Braunschweig, Germany) under the number DSM 2803.

Soon after its discovery, several Btt based plant protection products were placed on the market in the US, Canada, and Europe (Gelernter, 2004). One of these commercial products was Novodor^®^, containing the production strain NB125 (DSM 5526), a subculture of BI 256-82, which was registered in 1990 to control the Colorado potato beetle (*Leptinotarsa decemlineata*) (Gelernter, 2004). By Gamma-irradiation of NB125, NB176-1 (DSM 5480) was produced, which differed from NB125 by (i) oligosporogeny, (ii) a more than twofold increase of Cry3Aa protein production and crystals up to more than five times bigger than the crystals produced by NB125, and hence an increased insecticidal activity (Adams et al., 1994; Gurtler and Petersen, 1994). Based on cloning of the *cry3Aa* gene and Southern hybridization it had been proposed that a transposition-mediated duplication of the *cry3Aa* gene caused the Cry3Aa overproduction (Adams et al., 1994). The radiation-derived NB176-1 (DSM 5480) became eventually the new basis of the commercial product Novodor^®^ FC.

Recently, genomic data of NB176 (Novodor^®^ FC) has been published (Biggel et al., 2021), though its genome and number of plasmids have not been fully resolved, probably due to collapsing repeat sequences during the assembly process (Ekblom and Wolf, 2014; Fadeev et al., 2016; Arredondo-Alonso et al., 2017; Smits, 2019).

Here, we report the full genomes of the three Btt strains NB125 (DSM 5526), NB176-1 (DSM 5480) and the current production strain NB176, formulated in the plant protection product Novodor^®^ FC. The sequences were obtained by applying a hybrid *de novo* assembly approach using a combination of short (Illumina) and long (Nanopore) sequencing reads (Wick et al., 2017b). Comparisons of the three genomes allowed following and understanding the genomic rearrangements that had taken place from the former (parental strain before radiation) to the current production strain used as biocontrol agent (European Food Safety Authority, 2013).

The entomopathogenic Bt and their subspecies are members of the *Bacillus cereus sensu lato* (*s.l.*) group which also contains vertebrate pathogens, such as *B. cereus sensu stricto* (*s.s.*), *B. anthracis*, *B. weihenstephanensis* and others (Helgason et al., 2000; Liu et al., 2015; Ehling-Schulz et al., 2019).

Human pathogenicity of *B. cereus* is expressed as food poisoning of the emetic (vomiting) or diarrheal type (Turnbull et al., 1979; Schoeni and Wong, 2005; Rajkovic et al., 2008; Stenfors Arnesen et al., 2008; Messelhäußer and Ehling-Schulz, 2018). Whereas genes encoding the emetic toxin cereulide (CES) are absent from Bt strains (Ehling-Schulz et al., 2006; EFSA Panel on Biological Hazards, 2016; Bonis et al., 2021), diarrheal toxin genes encoding hemolysin BL (HBL), non-hemolytic enterotoxin (NHE), and cytotoxin K (CytK) have been demonstrated to be present on the chromosomes of several Bt subspecies (Prüß et al., 1999; Swiecicka et al., 2006; Ngamwongsatit et al., 2008; Bonis et al., 2021). However, there is little to no evidence that Btt or other commercial Bt strains produce such toxins in relevant enterotoxic levels (Damgaard, 1995; Raymond and Federici, 2017; Johler et al., 2018; Raymond and Federici, 2018). Since the use of Btt is for biocontrol purposes, a further focus of our study laid on the identification of such enterotoxin genes as well as on antimicrobial resistance (AMR) genes.

## 2 Materials and methods

### 2.1 Bacterial strains and cultivation

The parental NB125 (DSM 5526) and radiation-derived strain NB176-1 (DSM 5480) were obtained as freeze-dried cultures from the DSMZ (Leibniz Institute DSMZ-German Collection of Microorganisms and Cell Cultures GmbH, Braunschweig, Germany), whereas the current production strain NB176 (formulation Novodor^®^ FC) was provided as glycerol stock (vegetative cells in 1 ml glycerol) by Valent BioSciences (Libertyville, Illinois, United States) (**Table 1**).

**Table 1:** *Bacillus thuringiensis *subsp. *tenebrionis* (Btt) strains used for whole genome sequencing.

Strain notation	Deposit date	Received as/in	Provider	Reference
NB125 (DSM 5526)	Sept. 14, 1989	freeze-dried	DSMZ^1^	(Adams et al., 1994; Gurtler and Petersen, 1994)
NB176-1 (DSM 5480)	Aug. 10, 1989	freeze-dried	DSMZ	(Adams et al., 1994; Gurtler and Petersen, 1994)
NB176^2^		glycerol	Valent BioSciences	(European Food Safety Authority, 2013)

^1^DSMZ, Leibniz Institute DSMZ-German Collection of Microorganisms and Cell Cultures GmbH (Braunschweig, Germany). ^2^Lot #1979.

After receiving the three different Btt strains, they were always handled under sterile conditions to avoid any contamination. Glassware and culture media were autoclaved prior to use and all steps were performed under a sterile workbench. The freeze-dried strains NB125 and NB176-1 were received in tightly sealed glass vials and were handled according to the protocol of the DSMZ. In brief, the glass vials were broken open and the dried strains were mixed with 0.5 ml LB medium (1% tryptone, 0.5% yeast extract, 0.5% NaCl, pH 7.0 ± 0.2; Carl Roth, Karlsruhe, Germany). After 30 min rehydration, 0.25 ml of the suspension were used to inoculate 5 ml liquid LB medium. To recover NB176, the frozen glycerol stock was stabbed using a sterile loop and 5 ml liquid LB medium were directly inoculated. All bacterial cultures were grown at 30 °C with shaking at 200 rpm overnight.

### 2.2 Total genomic DNA extraction and whole genome sequencing

Starting from an aliquot of 1 ml overnight culture, total genomic (chromosomal + plasmid) DNA (gDNA) was extracted using the Wizard^®^ Genomic DNA Purification Kit (Promega, Madison, WI, USA), following the protocol for gram-positive bacteria. For cell lysis, the bacteria were treated with lytic enzyme in a final concentration of 2 mg/ml lysozyme (Sigma-Aldrich, St. Louis, Missouri, USA) for 60 min at 37 °C. After DNA precipitation, the gDNA was dissolved in 100 μl dH_2_O and stored at -80 °C until further use. The quality of gDNA was verified by measuring the absorbance ratios 260/280 and 260/230 using a Nanodrop 2000c spectrophotometer (Thermo Fisher Scientific, Wilmington, DE, USA). Concentration of gDNA was determined with a Quantus Fluorometer (Promega, Madison, WI, USA) and integrity was confirmed by TAE gel electrophoresis (0.8% agarose).

The identical stocks of gDNA were used for both Illumina (short read) and Nanopore (long read) sequencing. Short-read genomic data was obtained from approximately 500 ng gDNA per sample using a 150 bp paired-end protocol on an Illumina NextSeq 2000 sequencer (StarSEQ GmbH, Mainz, Germany). The genome size was estimated with 6 Mbp and between 6 to 7 M reads were ordered to achieve an estimated 150 to 175-fold read depth. The quality of the raw sequencing data was validated with FastQC (v0.72) (Andrews, 2010) and adapter and quality trimming (Phred quality score ≥ 30; minimum read length after trimming = 20 nt) was performed using Trim Galore! (v0.6.3.) (Krueger, 2015).

For long-read sequencing, the MinION sequencer (Oxford Nanopore Technologies, Oxford, UK) was utilized. Extracted gDNA was concentrated to approximately 30 ng/µl by vacuum evaporation using an Eppendorf Concentrator plus (Eppendorf, Hamburg, Germany) with the following options: 30 °C, brake = off and V-AQ. Multiplexing and library construction were performed using the Native Barcoding Expansion Kit (EXP-NBD104) and Ligation Sequencing Kit (SQK-LSK109), following the manufactures instructions with minor modifications: To increase DNA recovery, freshly prepared 80% ethanol was used throughout the protocol. Incubation times for gDNA binding to and elution from magnetic beads were extended to 15 min. All elution steps were conducted at 37 °C. After adapter ligation, large DNA fragments longer than 3 kbp were enriched using the Long Fragment Buffer (LFB), provided by the kit. The prepared library, consisting of three barcoded DNA samples, was loaded and sequenced on a MinION R9.4.1 flow cell (Oxford Nanopore Technologies Ltd., Oxford, UK) for 68 hours to receive approximately 5 M reads (15.76 Gb). Basecalling, demultiplexing and adapter trimming of long nanopore reads were performed using Guppy (v4.3.4). Subsequently, reads were filtered on minimum read quality (Q = 10) and minimum read length (≥1000 nt) using NanoFilt (v2.3.0) (De Coster et al., 2018). For each sample, basic statistics of long read sequencing data before and after filtering were calculated with NanoStat (v1.1.2.) (De Coster et al., 2018).

### 2.3 Hybrid *de novo* genome assembly

For the assembly of the Btt genomes (chromosomes and plasmids) the Unicycler tool (v0.4.8) was used, a SPAdes algorithm based genome assembler with varying k-mer sizes (Wick et al., 2017b). Default parameters and normal bridging mode were selected as settings for the Unicycler, using the quality filtered and adapter trimmed short (Illumina) and long read (MinION) sequencing data. The assembled genomes were visualized with Bandage (v0.8.1.) (Wick et al., 2015) and were further evaluated using QUAST (v5.0.2.) (Gurevich et al., 2013). To resolve the entire genomic structure, manual multiplicity adjustments were required for both NB176-1 and NB176 due to large inter-plasmidic repeats. Briefly, the best SPAdes graphs obtained from the hybrid *de novo* assemblies were visualized in Bandage. Subsequently, regions in question (e.g. assembled linear plasmid contig) were BLAST searched in Bandage and multiplicity calls were inspected using Unicycler’s graph color scheme (green = single-copy, yellow = two-copy, orange = three-copy, red = multi-copy). In regions where mis-assemblies have occurred, the multiplicities were adjusted manually following the protocol of the Unicycler webpage (https://github.com/rrwick/Unicycler/wiki/Multiplicity-mistake). The modified best SPAdes graph was then used as input for a follow-up hybrid assembly using Unicycler. For NB176 further manual curation was needed, since two plasmids collapsed into a shared repeat sequence, although both plasmids sequences were available in Unicycler’s final assembly graph. To finish the assembly and separate the two plasmids based on read depth, contigs were extracted and merged according to their graph path. Each completed assembly was further polished with the integrated tool Pilon (v1.20) (Walker et al., 2014) using the paired, highly accurate Illumina short reads.

### 2.4 Evaluation of genome assemblies

The quality of the assembly was evaluated by mapping paired Illumina reads back to the *de novo* assembled genome using BWA-MEM (Li and Durbin, 2009; Li, 2013). Proportion of mapped and unmapped reads was calculated for each strain using samtools (v1.9.). To examine the reads, which did not map to their respective assembly, unmapped paired-end reads were extracted from BWA-MEM output and *de novo* assembled into contigs. Taxonomy was assigned to each contig by blastn search against the nt NCBI database.

Coverage and depth of mapping (mean ± standard deviation [SD]) were calculated for each replicon (chromosome and plasmids) within each strain. Coverage plots were visually inspected in Geneious Prime (v2021.2.2) (Biomatters Inc., San Diego, CA, USA) to detect ambiguous regions in the assembly indicating possible errors in the assembled sequences. Circularity of replicons was verified by selecting reads spanning from both contig ends.

To assess assembly completeness in terms of gene content, BUSCO (v5.2.2.; genome mode) (Simão et al., 2015) with lineage-specific ortholog set for Bacillales was used (bacillales_odb10, 2021-02-23). Missing and duplicated BUSCO genes were reported, as they may not be of technical but biological origin such as gene loss and gene duplication.

### 2.5 Multiple sequence alignment and verification of nucleotide deletions

Multiple sequence alignment (MSA) of NB125, NB176-1 and NB176 was performed using Mauve progressive algorithm (Darling et al., 2010) in Geneious Prime (v2021.2.2). Each replicon (bacterial chromosome, plasmids) was considered individually and compared between all strains carrying that replicon. After MSA, indels were left realigned manually to correct gaps introduced by the aligner. To compare replicon sequences, sequence identity was calculated. Here, sequence identity [%] was defined as the percentage of bases that are identical in an alignment. For bacterial chromosome comparison, alignment was performed twice: An additional sequence identity analysis was performed after excluding a chromosomal deletion from the alignment. To verify detected nucleotide deletions in genome assemblies, paired Illumina reads were mapped with BWA-MEM against the *de novo* assembled genome sequence of the parental NB125. Samtools depth (Galaxy version 1.9) was used to compute the read depth at each genomic position and the output file was loaded in R (v4.1.2.) using RStudio (v2021.09.2) to create a coverage plot for the corresponding replicon.

### 2.6 Genome annotation and homology analysis of predicted coding sequences

Automatic annotation of protein coding DNA sequences (CDS) and RNA genes, transfer RNA (tRNA), ribosomal RNA (rRNA) as well as transfer messenger RNA (tmRNA), was performed using Prokka software (v1.14.6, default settings) (Seemann, 2014). Ambiguities in the chromosome annotation between the three strains were identified by aligning the entire chromosome including annotations with Mauve progressive algorithm in Geneious. One CDS in NB176 was not annotated automatically although the complete sequence was available (100% sequence identity). To adjust the annotation, the CDS was added manually.

In the next step, annotated gene sets (GFF3) were used for homology analysis of CDS: Each replicon (bacterial chromosome, plasmids) was considered individually and the predicted CDS were compared between all strains carrying that replicon. To identify CDS homologies, the fully automated Roary software (v3.13, default settings, minimum percentage amino acid identity = 95%, split-paralogs, 100% core genes threshold) was used (Page et al., 2015). Occurring paralogous genes were analyzed individually (paralog splitting) to investigate not only sequence homologies but also their genomic position. The number of shared (= present in all three strains), variable (= present in two strains) and unique (= present in a single strain) CDS were determined. Following this computer based CDS comparison, detected homologies and CDS identified as variable or unique were verified manually. Gene presence/absence files created by Roary software were further analyzed in R (v4.1.2.) using RStudio (v2021.09.2).

Since all annotated features (CDS + RNA genes) were numbered consecutively by Prokka software, the location and annotation of RNA genes were also included in the final gene presence/absence files (**Supplementary Tables 1**–**8**). Homology of RNA genes was not investigated.

### 2.7 Analysis of CDS located on a chromosomal deletion

It was assessed if copies of the CDS located on a deleted stretch of chromosomal DNA can be found somewhere else in the corresponding genome. The set of deleted CDS was extracted and batch searched against the corresponding genome using MEGABLAST (default settings, i.e. max. E-value = 0.05, word size = 28) in Geneious Prime. MEGABLAST results were sorted into two bins (hit vs. no hit) and identified hits were batch searched again to obtain a hit table with nucleotide identity and coverage values. Only CDS that were found to cover at least 70% of the BLAST searched CDS were considered as present. The analysis was carried out at the nucleotide level, since there is no comparable MEGABLAST for protein searches available.

To classify CDS that were located on the deletion site into functional categories, corresponding Clusters of Orthologous Groups (COG) identifiers were used. COG numbers were extracted from Prokka annotations and subsequently searched in the NCBI COG database. The total number of CDS in each detected COG class was counted to investigate overrepresented COG categories. If a CDS was assigned to more than one COG category, a 1 was added to the count of each respective COG class.

### 2.8 *In silico* prediction of virulence and resistance genes and sequence typing

Assembled genomes were submitted to BTyper software (Galaxy version 2.0.3, default settings) (Carroll et al., 2017) to identify virulence genes. The software uses a search/comparison based approach (default cutoffs: minimum nucleotide identity = 50%, minimum query coverage = 70%) against the BTyper virulence database, containing 44 virulence genes specific for the *B. cereus* group.

Besides virulence gene prediction, BTyper was used for *in silico* multi-locus sequence typing (MLST), *panC* clade assignment, and *rpoB* allelic typing. To determine the genome position of all sequences detected by BTyper software, the corresponding reference amino acid (aa) or nucleotide (nt) sequences were downloaded and tblastn (aa) or blastn (nt) searched in Geneious Prime. Location of BLAST hits and corresponding annotation IDs of strain NB125 were reported. Homologous genes (and their annotation IDs) for NB176-1 and NB176 can be found in the corresponding annotation tables (**Supplementary Tables 1**, **8**).

Antimicrobial resistance genes were predicted using the software ABRicate (Galaxy Version 1.0.1; (Seemann, 2018)), including the ARG-ANNOT (2022-Jun-13, n = 1749; (Gupta et al., 2014)), ResFinder (2022-Jun-13; n = 3144; (Zankari et al., 2012)), CARD (2022-Jun-13; n = 2220; (Jia et al., 2016)), and NCBI AMRFinderPlus (2022-Jun-13; n = 6146; (Feldgarden et al., 2019)) databases. ABRicate was used with an 80% nucleotide identity and 70% coverage threshold. In addition, the tool AMRFinderPlus (Galaxy Version 3.1.1b+galaxy1; (Feldgarden et al., 2021), database version 2022-05-26.1) was run in combined mode (nucleotide + protein) allowing the use of Hidden Markow Models (HMM) to enhance sensitivity for novel AMR genes. The default settings (“minimum identity for a blast-based hit =-1.0, means use a curated threshold if it exists and 0.9 otherwise, minimum coverage of the reference protein = 0.5”) were used to allow the use of manually curated BLAST cutoffs.

### 2.9 Phylogenetic analysis

The complete genome sequences of NB125, NB176-1 and NB176 were uploaded to the Type (Strain) Genome Server (TYGS) for a whole genome-based taxonomic analysis (Meier-Kolthoff and Göker, 2019). Closest type strain genomes were determined automatically (**Supplementary Table 11**). All pairwise comparisons between the three uploaded strains as well as between the three strains and closest related type strains were performed using the Genome Blast Distance Phylogeny (GBDP) approach. Accurate intergenomic distances were inferred under the algorithm “trimming” and distance formula d_5_ (Meier-Kolthoff et al., 2013). One hundred distance replicates were calculated each. Digital DNA-DNA-Hybridization (dDDH) values and confidence intervals were calculated using the recommended settings of the GGDC 3.0 (Meier-Kolthoff et al., 2013; Meier-Kolthoff et al., 2022). Based on the intergenomic distances, a balance minimum evolution tree with branch support was inferred *via* FASTME 2.1.6.1., including Subtree-Pruning-Regrafting (SPR) postprocessing (Lefort et al., 2015). The tree was rooted at the midpoint (Farris, 1972) and visualized with PhyD3 (Kreft et al., 2017). Branch support was inferred from 100 pseudo-bootstrap replicates each.

The automatically determined closest type strain genomes were downloaded from NCBI and subsequently uploaded to the JSpeciesWS webserver (Richter et al., 2016) for ANIb (Average nucleotide identity algorithm using BLAST) calculation (Goris et al., 2007). Pairwise ANIb values between the selected type strain genomes and the three genomes under assessment were calculated. The average of the two reciprocal ANIb values was calculated to provide a single ANIb value for each genome pair.

## 3 Results

### 3.1 Sequencing statistics

The output from the Illumina platform was 6.4 M (NB125), 6.9 M (NB176-1), and 7.2 M (NB176) reads with an average Phred quality score of Q = 33, corresponding to a sequencing accuracy of >99.9% (**Table 2**). After adapter removal, quality trimming and read pairing the number of reads was reduced by approximately 2% (**Table 2**). Long read sequencing (MinION) yielded in 1.2 M (NB125), 2 M (NB176-1) and 896 K (NB176) reads with a length ranging between N50 = 4.4 kbp (NB176-1) to N50 = 6.4 kbp (NB125) (**Table 2**). The number of long reads was reduced by 35-40% per sample after filtering by quality (Q ≥ 10) and length (≥ 1000 nt) (**Table 2**).

**Table 2:** Sequencing summary table for parental strain NB125 and derived strains NB176-1 and NB176.

Strain	Short read sequencing (Illumina)		Long read sequencing (MinION)			
	Total reads	No. quality filtered reads ^a^	Total reads ^b^	No. quality and length filtered reads ^c^	N50 (nt)	Run time (h)
NB125	6,390,134	6,252,064	1,203,211 (A)	792,822	6,400	68
NB176-1	6,925,308	6,765,952	2,009,229 (A)	1,194,730	4,413	68
NB176	7,221,370	7,063,050	896,236 (A)	557,330	4,904	68

a TrimGalore!: adapter removal and quality trimming (Phred quality score ≥ 30; minimum read length after trimming = 20 nt), output of paired reads only.

b Same letter indicates that samples were run simultaneously with different barcodes on the same flow cell.

c Nanofilt: Minimum read quality Q = 10, minimum read length = 1000 nt.

### 3.2 Hybrid assemblies and evaluation

To resolve the entire genome of NB125, NB176-1 and NB176, a hybrid *de novo* assembly based on short and long read sequencing data was performed. This approach resulted in sufficient high-quality reads to allow the separation of chromosomal and plasmid sequences and their complete reconstruction (**Tables 3**, **4**). The genome length was found to vary between 6 to 6.3 Mbp with an identical GC content of 35.1% (**Table 3**). The chromosomes were 5.4 to 5.6 Mbp long and in each strain, the chromosome was associated with six putative plasmids that were between 14.9 to 250.5 kbp in length (**Tables 3**, **4**). The plasmid sequences were called putative plasmids (ppl) since their identification method was sequence based. In the process of whole genome reconstruction, the Unicycler tool resolved the genome of NB125 entirely, without any manual adjustment. The assembled contigs of NB125 chromosome (5.6 Mbp) as well as its six plasmids (250-ppl, 185-ppl, 137-ppl, 68-ppl, 43-ppl and 14-ppl) were circular and covalently closed (**Tables 3**, **4**). For NB176-1 and NB176, the reconstruction was hampered by inter-plasmidic repeats and required manual adjustment by fixing ambiguous repeat regions with the help of multiplicities. Here, the chromosomes were found to be 5.6 and 5.4 Mbp long for NB176-1 and NB176, respectively (**Table 3**). The plasmid set up for NB176-1 and NB176 was slightly different from NB125: 250-ppl, 185-ppl, 99-ppl, 68-ppl, 43-ppl and 14-ppl (**Table 4**).

**Table 3:** Assembly statistics of Btt strains NB125, NB176-1, and NB176. Total length [bp] and GC content [%] of the whole genome (chromosome + plasmids) and the single bacterial chromosome as well as the total number of plasmids are given.

Strain	Genome		Chromosome		No. plasmids
	Length [bp]	GC [%]	Length [bp]	GC [%]	
NB125	6,305,941	35.1	5,605,440	35.3	6
NB176-1	6,268,965	35.1	5,606,443	35.3	6
NB176	6,090,597	35.1	5,428,075	35.4	6

**Table 4:** Sequence based detection of seven putative plasmids (ppl) found in NB125, NB176-1 and NB176.

Plasmid	Length [nt]	GC [%]	Presence in Btt strain			No. CDS	No. tRNA	Minimum pairwise nt identity (%) **
			NB125	NB176-1	NB176			
250-ppl	250,492	33.9	X	X	X	236/237*	1	>99.965
185-ppl	185,418	32.6	X	X	X	168	0	>99.995
137-ppl	137,412	31.7	X			168	0	–
99-ppl	99,433	32.4		X	X	109	0	100
68-ppl	68,504	32.2	X	X	X	78	0	100
43-ppl	43,822	35.4	X	X	X	56	0	100
14-ppl	14,853	31.0	X	X	X	30	0	100

The number of coding sequences (CDS) and tRNAs were detected by automated annotation.

*236 CDS annotated for NB176-1; 237 CDS annotated for NB125 and NB176.

**A detailed pairwise comparison can be found in the supplementary data (**Supplementary Table 12**).

To assess the quality of the genome assemblies, paired Illumina reads were mapped against their corresponding whole genome assembly. Here, 99.71%, 99.73% and 99.86% of all Illumina reads of NB125, NB176-1 and NB176 mapped, respectively. To investigate the origin of unaligned reads, unmapped, paired-end reads were extracted, *de novo* assembled into contigs and taxonomy was assignend to each contig by BLAST search against the NCBI database. For each NB125 and NB176-1, two contigs were *de novo* assembled from unmapped paired-end reads. These contigs were assigned to cattle chromosomes (*Bos gaurus*, *Bos taurus*), most likely due to culture media: Prior to freeze-drying, the two strains deposited at DSMZ were cultured on media containing beef-extract. For NB176 it was not possible to *de novo* assemble the unmapped, paired-end reads into contiguous sequences. Therefore, 0.14% unassigned reads were found for NB176.

Another evaluation step comprised the identification of 450 bacillales core genes using BUSCO software leading to a completeness score of 99.8% (98.7% single copy, 1.1% duplicated and 0.2% missing) for NB125, NB176-1 and NB176. In all three strains, the tryptophan repressor (BUSCO ID 148693at1385) was missing and five BUSCO genes were duplicated (**Table 5**). Both copies of four duplicated BUCSO genes were located on the chromosome (**Table 5**), whereas *recA* was located on the chromosome and on 43-ppl.

**Table 5:** Duplicated BUSCO genes in NB125, NB176-1, and NB176 according to analysis of 450 core genes listed in the bacillales_odb10, (2021-02-23) dataset.

BUSCO ID	ID_NB125_	Name of encoded protein
32106at1385	2498 + 5192	translocase subunit SecY
34686at1385	1426 + 2913	aspartate-semialdehyde dehydrogenase
63007at1385	4106 + 4171	ferrochelatase
76591at1385	2286 + 3754	chorismate synthase
50377at1385	1446 + 10*	RecA**

BUSCO ID and the corresponding name of the encoded protein are given. The annotation ID for NB125 is provided only. Homologous genes (and their IDs) for NB176-1 and NB176 can be found in the corresponding annotation table (**Supplementary Tables 1**, **3**).

*ID of plasmid 43-ppl.

**One copy was located on the chromosome, and the other copy was located on 43-ppl plasmid.

### 3.3 Sequence comparison

**Figure 1:**
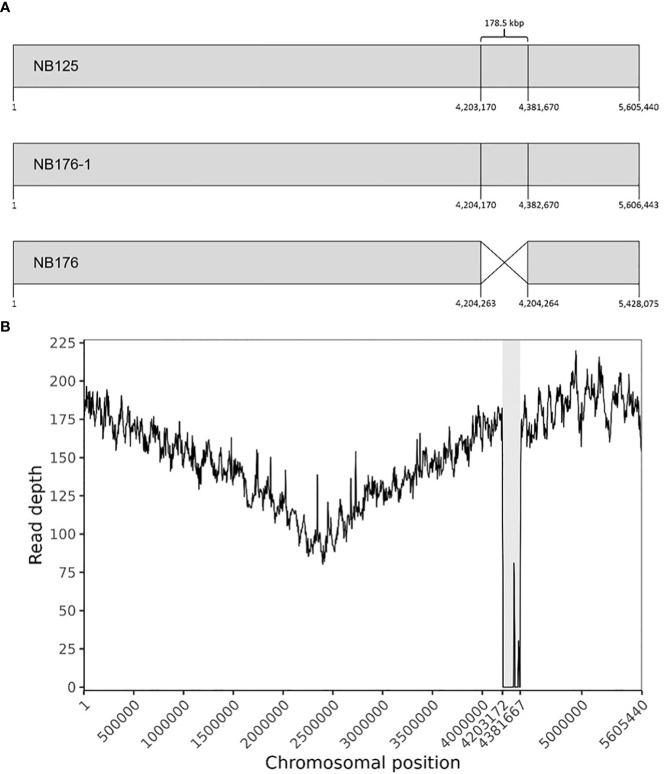
**(A)** Schematic linear presentation of the circular chromosomes of NB125, NB176-1, and NB176 (grey boxes). Chromosomal positions are given for each strain below. NB176 contains a 178.5 kbp deletion between positions 4,204,263 and 4,204,264. **(B)** Chromosome coverage plot of NB176. Short reads of NB176 were mapped against the genome reference sequence of parental NB125. The drop in the sequencing depth between position 4,203,170 and 4,381,670 verified the detected chromosomal deletion of ~178.5 kbp (highlighted in grey) in NB176. Read depth was averaged every 5000 bp along the reference genome.

The chromosome of NB176-1 was 1003 bp longer than that of NB125, but they shared an identical GC content (35.3%, **Table 3**). The chromosome of NB176 was shorter (~178.5 kbp) than those of NB125 and NB176-1 and had a slightly higher GC content of 35.4% (**Table 3**). Multiple sequence alignment revealed a chromosomal deletion of 178,499 bp in NB176 (**Figure 1A**). To verify the detected deletion, short reads of NB176 were mapped against the *de novo* assembled genome sequence of the parental NB125 where a drop to zero in read depth between 4,203,170 and 4,381,670 confirmed the detected deletion (**Figure 1B**). MSA was also used to compare the three chromosomes at the nucleotide level: The nucleotide sequence identity of NB125, NB176-1, and NB176 was above 96% and increased to >99.95% after excluding the deletion site from the alignment (**Supplementary Table 13**).

**Figure 2:**
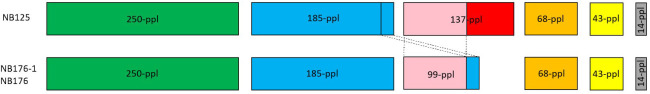
Schematic presentation of the six plasmids of NB125, NB176-1, and NB176. Each circular plasmid is linearized and represented in scale by a rectangle. Same colors represent homologous regions. Plasmid 137-ppl has a unique region (red) and a region of 72,351 nt (pink) that is shared with 99-ppl (100% nt sequence identity). In addition to that shared region, a shorter part of 99-ppl (27,082 nt) is also found in 185-ppl (blue, 100% nt sequence identity), located on an inter-plasmidic repeat region with a total length of 28,743 nt. This duplicated region is carrying the *cry3Aa* gene, which thereby is present in two copies, on 99-ppl and 185-ppl, for NB176-1 and NB176, and only in one copy for NB125. Besides this recombination, a deletion of a single stretch of plasmid DNA (65,061 nt) compared to 137-ppl of the parental strain NB125 was detected in NB176-1 and NB176.

Six circular plasmids (14-ppl, 43-ppl, 68-ppl, 137-ppl, 185-ppl, 250-ppl) were reconstructed for strain NB125, ranging in length and GC content from 14.9 kbp to 250.5 kbp as well as 31% (14-ppl) to 35.4% (43-ppl) (**Table 4**). For both NB176-1 and NB176, six circular plasmids (14-ppl, 43-ppl, 68-ppl, 99-ppl, 185-ppl, 250-ppl) were assembled. These plasmids were identical in their length and GC content (**Table 4**). The difference between the two sets of plasmids was due to the presence of 137-ppl in NB125 and 99-ppl in NB176-1 and NB176 (**Table 4**). Multiple sequence alignment revealed that 99-ppl is a recombinant plasmid consisting of two parts: One larger part (72,351 bp) was homologous to 137-ppl of NB125 (100% nt sequence identity) and a shorter part (27,082 nt) that was also found in 185-ppl (100% nt sequence identity), located on an inter-plasmidic repeat region with a total length of 28,743 nt (**Figure 2**). This duplicated region is carrying the *cry3Aa* gene, encoding the coleopteran-specific protein Cry3Aa (Höfte et al., 1987). Therefore, *cry3Aa* is present in two copies, on 99-ppl (ID_99-ppl_ = 109) and 185-ppl (ID_185-ppl_ = 168) for the derivative strains NB176-1 and NB176, and only in one copy for NB125 (185-ppl). Moreover, a deletion of a single stretch of plasmid DNA (65,061 nt) compared to 137-ppl of the parental strain NB125 was detected in NB176-1 and NB176.

In addition to the *cry3Aa* gene, two other pesticidal genes, encoding for Mpp23Aa1 (ID_185-ppl_ = 8; 100% aa identity/100% coverage to GenBank accession no. AAF76375) and Xpp37Aa1 (ID_185-ppl_ = 7; 100% aa identity/100% coverage to GenBank accession no. AAF76376) were found on the original Cry3Aa encoding plasmid 185-ppl.

### 3.4 Genome annotation

For the chromosome of NB125, NB176-1 and NB176, a total number of 5760, 5761 and 5579 annotations were predicted, respectively. The majority of 5610 (NB125), 5611 (NB176-1) and 5429 (NB176) annotations were identified as CDS, whereas the remaining 150 annotations were assigned to RNA genes (**Table 6**). A total number of 30, 56, 78, 109, and 168 CDS were predicted for 14-ppl, 43-ppl, 68-ppl, 99-ppl, and 137-ppl, respectively (**Table 4** and **Supplementary Tables 2**–**6**). For 185-ppl a total number of 168 CDS was predicted for each strain, although nucleotide sequences were slightly different (**Table 4** and **Supplementary Tables 7**, **12**). Homology analysis of CDS (homology aa cutoff ≥95%) revealed that there were no differences in gene content. In addition, order and orientation of predicted CDS was identical for 185-ppl. A total number of 237 (NB125, NB176) and 236 (NB176-1) CDS as well as one tRNA gene was found for 250-ppl (**Table 4** and **Supplementary Table 8**). According to the computer-based comparison of CDS, 236 CDS were found to be homologous in all strains. In 250-ppl of NB176-1, a nucleotide difference caused the stop codon to be skipped in CDS ID_250-ppl/NB176-1_ = 66. Thus, CDS no. 66 of NB176-1 spans the length of the two CDS no. 66 and no. 67 of NB125 and NB176 (ID_250-ppl/NB125/NB176_ = 66 + 67, **Supplementary Table 8**).

**Table 6:** Annotation statistic for each final chromosome assembly of Btt strain NB125, NB176-1, and NB176.

Genomic Feature	NB125	NB176-1	NB176
CDS	5610	5611	5429*
rRNA	42	42	42
tRNA	107	107	107
tmRNA	1	1	1
Total no. annotations	5760	5761	5579

*One CDS (ID_NB176_ = 4935*) was not annotated automatically and added manually.

Prediction of CDS and RNA (rRNA, tRNA, tmRNA) genes was performed using Prokka software.

**Figure 3:**
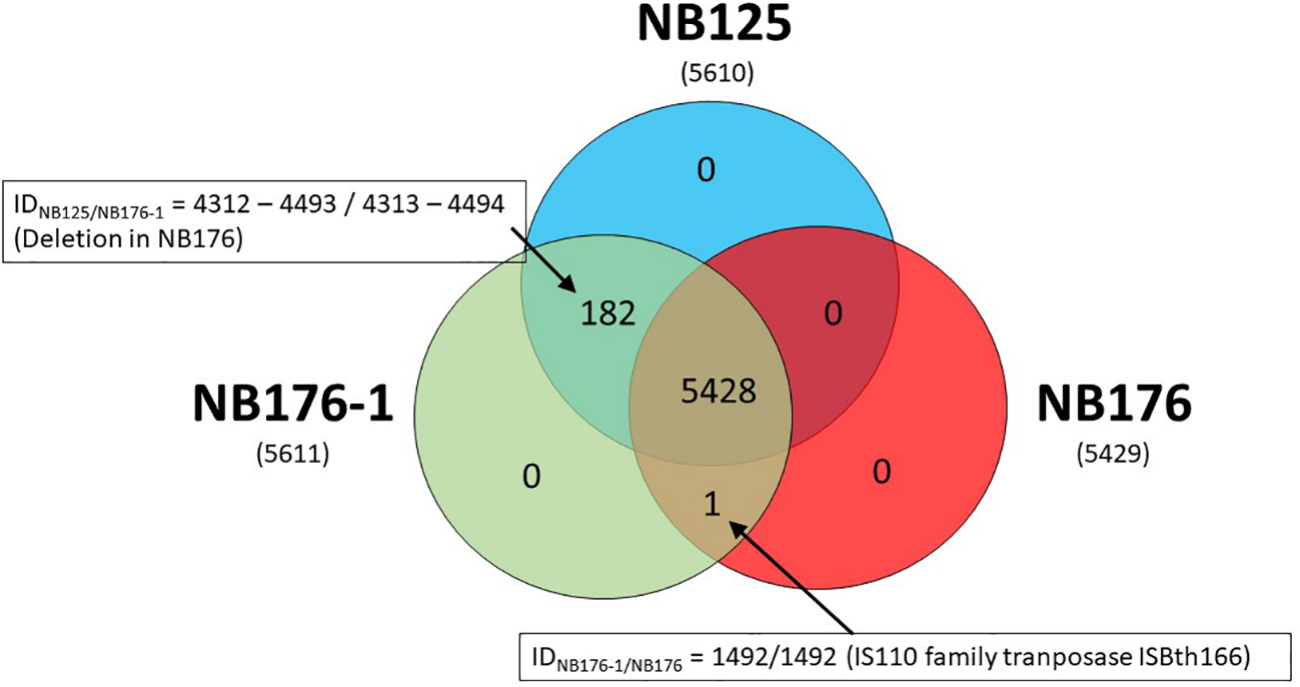
Shared, variable and unique chromosomal CDS detected for parental strain NB125 and its derived strains NB176-1 and NB176. In total, 5611 CDS were detected, of which 5428 were shared by all three strains. The number of chromosomal CDS predicted for each strain are given in parenthesis below the strain names. Numbers inside the Venn diagram represent unique, variable and shared CDS.

Coding sequences of the three annotated chromosomes were compared using an automated approach based on Roary software with a homology criterion of ≥95% amino acid sequence identity. This automated method was verified manually and adjusted when required. Based on this approach, a set of 5428 chromosomal CDS was found to be homologous for all three strains NB125, NB176-1, and NB176 (=shared genes) (**Figure 3**). The main differences in chromosomal CDS were the absence of 182 CDS (ID_NB125_ = 4312-4493) in NB176 due to the chromosomal deletion of 178.5 kbp, and one additional IS110 family transposase ISBth166 gene (ID_NB176-1_ = 1492 and ID_NB176_ = 1492) that was present in NB176-1 and NB176, but not in the parental NB125 (**Figure 3**). Initially, two CDS were reported to fail the homology criterion due to short nucleotide insertions/deletions (indels): The homologous CDS ID_NB125_ = 1544, ID_NB176-1_ = 1545 and ID_NB176_ = 1545 were located at the same relative position of the chromosomes. It was found that ID_NB176-1_ = 1545 differed from ID_NB125_ = 1544 and ID_NB176_ = 1545 by a 441 bp deletion resulting in the loss of 147 amino acids (aa) (aa identity = 78.1%) (**Table 7**). Similarly, the homologous CDS ID_NB125_ = 3195, ID_NB176-1_ = 3196 and ID_NB176_ = 3196 differed from each other that ID_NB176-1_ = 3196 and ID_NB176_ = 3196 had a 294 bp deletion resulting in the loss of 98 aa in the middle of the aa sequence, which reduced the overall aa identity to 92.3%. The remaining predicted aa of the CDS were identical between all strains (**Table 7**).

**Table 7:** Two sets of chromosomal CDS failing the homology criterion (≥95% amino acid sequence identity) of the fully automated detection of homologous CDS using Roary software.

Genomic feature	Prokka annotation no.			Length		Amino acid sequence identity
	ID_NB125_	ID_NB176-1_	ID_NB176_	nt	aa	
CDS	1544		1545	2013	670	78.1%
		1545		1572	523	
CDS	3195			3807	1268	92.3%
		3196	3196	3513	1170	

The software based approach identified ID_NB176-1_ = 1545 and ID_NB125_ = 3195 as unique genes due to their deletions. Manual verification identified these genes as homologous to CDS with deletions, present in the other two Btt strains, as well.

It was further assessed if the additional transposase gene (ID_NB176-1_ = 1492 and ID_NB176_ = 1492) occurs once, or whether it is an extra gene copy that occurs frequently in the bacterial chromosome. The results of a blastn search revealed that this gene occurs 13 times on the chromosome of the parental NB125 (8 copies with 100% nt identity and 100% coverage, and 5 copies with 97.6% nt identity/100% coverage), whereas it occurs 14 times on the chromosome of NB176-1 and NB176 (9 copies with 100% nt identity/100% coverage, and 5 copies with 97.6% nt identity/100% coverage). Therefore, both NB176-1 and NB176 carry one additional copy of this transposase gene.

**Figure 4:**
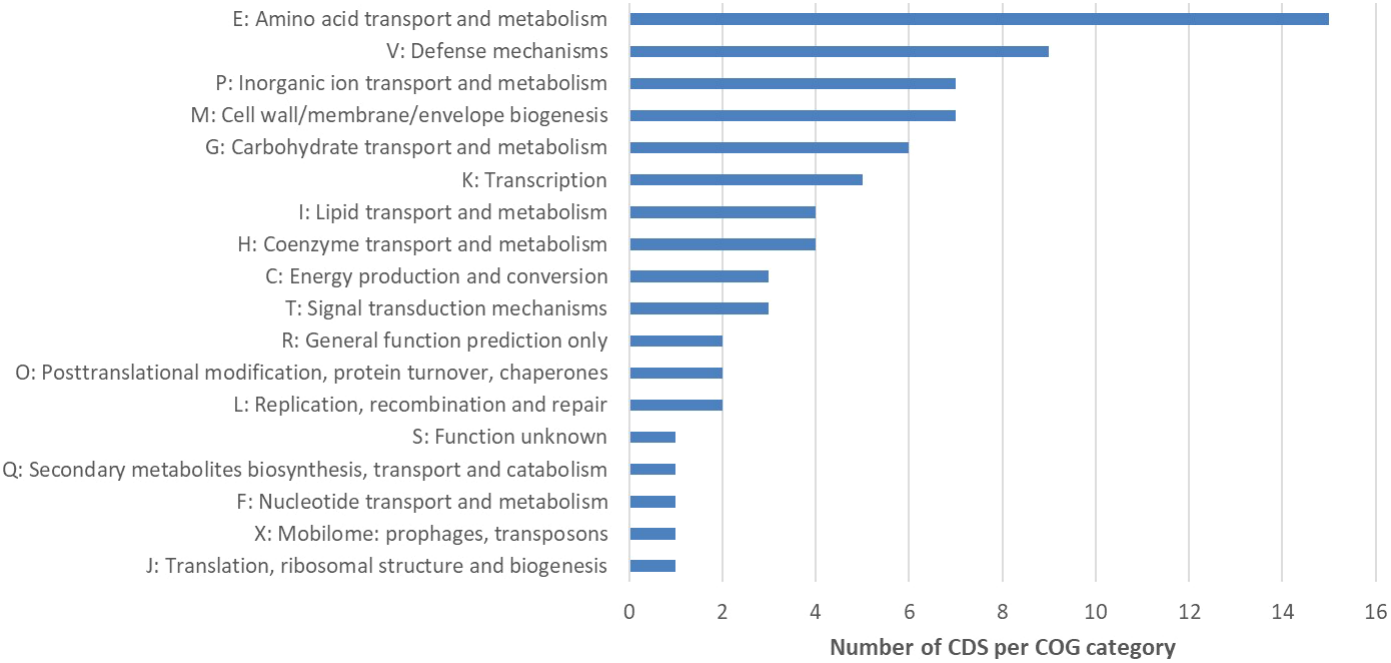
Cluster of Orthologous Groups (COG) analysis of CDS located within the deletion site of NB176. The number of CDS per COG category is given. A total of 182 predicted CDS were located on the chromosomal deletion in NB176. Eighty-nine CDS were annotated with the name of the encoded protein and sixty of these annotations included COG identifiers. These COG numbers were searched in the NCBI COG database to classify the 60 genes into 18 groups (16 functional groups + R = General function prediction only, + S = Function unknown). Due to assignments of CDS into multiple groups, 74 assignments were made. To investigate overrepresented COG categories, the total number of CDS in each detected COG class was counted. If a CDS was assigned to more than one COG category, a 1 was added to the count of each respective COG class.

In the next step, the automatic annotation of all 182 CDS located on the deletion site, was examined. From these, a total of 93 CDS (~51.1%) were annotated as hypothetical proteins (**Supplementary Table 9**). Eighty-nine CDS (~48.9%) were annotated with the name of an encoded protein and 34 (~38.2%) of these annotations included Enzyme Commission (EC) numbers (**Supplementary Table 9**). Sixty (~67.4%) out of 89 CDS were assigned to Cluster of Orthologous Groups (COG) identifiers (**Supplementary Table 9**). To classify these genes into functional categories, the corresponding COG identifiers were searched in the NCBI COG database. Genes located on the deletion were assigned to 16 functional groups (+ R = General function prediction only, + S = Function unknown) (**Figure 4**). After this functional assignment, the total number of CDS in each detected COG class was counted. Most genes were assigned to the COG category Amino acid transport and biosynthesis. As an example, the ABC transporter complex TcyABC involved in L-cystine import is located within the deletion site. The second largest COG category represented proteins with function in Defense mechanisms. Several genes associated with bacitracin resistance, e.g. a bacitracin efflux BceAB type ATP transporter were detected. In addition, some annotations without a COG identifier can be assigned to the function Defense mechanisms e.g. tetracycline resistance protein, class B; guanidinium exporter, and bacitracin transport ATP- binding protein BcrA (**Supplementary Table 9**). Further examples for CDS located at the deletion site were two putative ABC transporters YknXYZ (Cell wall/membrane/envelope biogenesis; Defense mechanisms), a Na(+)/H(+)-K(+) antiporter GerN associated with endospore germination (Inorganic ion transport and metabolism) and a licABC operon (Carbohydrate transport and metabolism), involved in the uptake and metabolism of lichenin degradation products (**Supplementary Table 9**).

To analyze, whether the 182 located within the deletion site, can be found somewhere else in the genome of NB176 the set of deleted CDS was extracted from the parental genome NB125 and batch searched against the NB176 genome using MEGABLAST algorithm. From the 182 CDS located on the chromosomal deletion, twelve CDS were found with hits elsewhere in the genome (**Table 8**). Four CDS were found with one homologous copy: ID_NB125_ = 4334, 4336, 4412, and 4417. Except for ID_NB125_ = 4336, which is located on 99-ppl, the CDS were located on the chromosome. For these four CDS, the nucleotide identity and CDS coverage ranged between 73.8% to 92.3% and 76.8% to 100%, respectively (**Table 8**).

**Table 8:** Coding sequences, located on the deleted region that were found elsewhere in the genome of NB176.

ID_NB125_	Annotation	Total number of hits	Replicon	Nucleotide id [%]/cov [%]	^1^ID_NB176_
4334	Type VII secretion system extracellular protein A	1	chr	92.3/100	3197
4336	IS3 family transposase ISBth10	1	99-ppl	73.8/76.8	41
4412	Bacitracin export ATP-binding protein BceA	1	chr	86.5/97.3	293
4417	Transcriptional regulatory protein WalR	1	chr	82.2/100	271
4420	IS4 family transposase ISBce10	4	chr	100/100	1476; 2530
			250-ppl	100/100	109
			185-ppl	100/100	138
4421	hypothetical protein	4	chr	100/100	1477; 2529
			250-ppl	100/100	*
			185-ppl	100/100	139
4422	hypothetical protein	4	chr	100/100	1478; 2528
			250-ppl		110
			185-ppl		140
4423	Bacitracin transport ATP-binding protein BcrA	4	chr	99.9/100	1479; 2527
			250-ppl	99.9/100	111
			185-ppl	99.9/100	141
4424	hypothetical protein	4	chr	100/100	1480; 2526
			250-ppl	100/100	113
			185-ppl	100/100	143
4425	Undecaprenyl-disphospatase BcrC	4	chr	100/100	1481; 2525
			250-ppl	100/100	114
			185-ppl	100/100	144
4426	IS3 family transposase ISBt2	3	chr	99.4/100	1465
			250-ppl	99.4/100	187
			99-ppl	93.9/99.8	61
4475	IS66 family transposase ISMma14	8	chr	100/100	4222; 4926; 5422; 5473
			250-ppl	100/100	190; 238
			185-ppl	100/100	45; 73

The corresponding IDs of NB176 are provided. In total, 12 out of 182 CDS were detected using MEGABLAST (default settings, i.e. max. E-value = 0.05, word size = 28), followed by post-processing using a query coverage cutoff of 70%.

^1^ID number belongs to the corresponding replicon. Further information is provided in the respective annotation tables of the bacterial chromosome (**Supplementary Table 1**), 250-ppl (**Supplementary Table 8**), 185-ppl (**Supplementary Table 7**), and 99-ppl (**Supplementary Table 5**).

^*^CDS was not automatically annotated. Hit start: 107,923; Hit end: 107,831.

The remaining eight CDS had more than one homologous CDS copy on the genome. Copies were found on the chromosome, 99-ppl, 185-ppl and 250-ppl. Except for ID_NB125_ = 4426 and 4475, CDS copies were found in duplicates on the chromosome and another two copies on 185-ppl and 250-ppl (**Table 8**). The nucleotide identity of the multi copy CDS varied between 93.9% to 100% and the coverage of the CDS was above >99.8% (**Table 8**). A block of six consecutive CDS (ID_NB125_ = 4420 to 4425) was found in the same order and orientation at four other locations in the genome of NB176. This block of genes occurred twice on the chromosome and once on 185-ppl and 250-ppl (**Table 8**). This repetitively occurring block of CDS carried *BcrA* and *BcrC* genes, both associated with bacitracin resistance.

In total, three out twelve reoccurring CDS were annotated as hypothetical proteins; four CDS were annotated as transposase genes, and three were associated with bacitracin resistance, In addition, one virulence gene associated with secretion and one gene annotated as a transcriptional regulatory protein was found to be located elsewhere in the genome (**Table 8**).

### 3.5 *In silico* prediction of virulence and antimicrobial resistance genes

The presence of 44 virulence genes specific for the *B. cereus* group was examined *in silico* by BTyper software (nucleotide identity ≥50%, coverage ≥70%). For NB125, NB176-1 and NB176 the same set of 23 virulence genes was predicted (**Table 9**) and the corresponding nucleotide sequences were identical. Some enterotoxins associated with diarrheal symptoms are expressed by operons: Both *nhe* (*nheABC*) and *hbl* (*hblABCD*) operon genes were identified in their entirety in the genomes of three analyzed strains (**Table 9**). However, the *nhe* promoter was disrupted by a transposase (ID_NB125_ = 3455, ID_NB176-1_ = 3456, ID_NB176_ = 3456) insertion in all three strains. Further, three putative enterotoxin genes encoding for the one-component proteins FM (e*ntFM*), T (*BceT*) and A (*entA*); two metalloprotease genes (*inhA1*, *inhA2*); and two genes (*clo*, *hlyII*) encoding for pore forming hemolysins (cerolysin O, hemolysin II) were detected. Three different phospholipase C genes were found; *plcA* encoding phosphatidylinositol-specific phospholipase C (PI-PLC), *cerA* encoding phosphatidylcholine-specific phospholipase C (PC-PLC), and *cerB* encoding sphingomyelinase (SM-PLC). PC-PLC and SM-PLC are encoded in an operon (*cerAB*) and form the biological complex cereolysin AB. Two genes coding for regulatory proteins, *hlyIIR*, regulating *hlyII*, and the pleiotropic regulator *plcR*, controlling the expression of multiple virulence genes, were found. Capsule genes (*hasA*, *cap*) and genes associated with the production of anthrax toxin (*pagA*, *lef*, *atxA*, *cya*) were not detected. In addition, all strains were negative for *ces* as well as *cytK*.

**Table 9:** Virulence genes specific for the *B. cereus* group predicted by BTyper software (minimum percent amino acid (aa) identity to reference sequence: 50%, minimum coverage of reference sequence = 70%).

Virulence gene	Virulence gene name	GenBank accession no. of reference aa sequence	^1^aa identity and coverage percentages (id [%]/cov [%])	Prokko no. ID_NB125_
*bceT*	diarrheal toxin [Bacillus cereus]	BAA04134.1	99.15/96.99	3191
*^2^cerA/plcB*	cereolysin A [Bacillus cereus]/phospholipase C [Bacillus cereus ATCC 14579]	AGL98059.1/NP_830483.1	100/100	4609
*cerB*	cereolysin B [Bacillus cereus]	AGL98063.1	94.86/93.39	4608
*clo*	cereolysin O [Bacillus cereus]	AAX88798.1	99.21/100	31
*entA*	enterotoxin/cell-wall binding protein [Bacillus cereus ATCC 14579]	NP_834902.1	94.97/100	5599
*entFM*	enterotoxin [Bacillus cereus ATCC 14579]	NP_831723.1	95.14/100	3379
*nheA*	non-hemolytic enterotoxin lytic component L2 [Bacillus cereus ATCC 14579]	NP_831582.1	99.74/100	3454
*nheB*	non-hemolytic enterotoxin lytic component L1 [Bacillus cereus ATCC 14579]	NP_831583.1	100/100	3453
*nheC*	enterotoxin C [Bacillus cereus ATCC 14579]	NP_831584.1	98.33/100	3452
*hblA*	hemolysin BL binding component precursor [Bacillus cereus ATCC 14579]	NP_832845.1	99.47/100	2126
*hblB*	hemolysin BL binding component precursor [Bacillus cereus ATCC 14579]	NP_832844.1	98.28/100	2127
*hblC*	hemolysin BL lytic component L2 [Bacillus cereus ATCC 14579]	NP_832847.1	98.18/100	2124
*hblD*	hemolysin BL lytic component L1 [Bacillus cereus ATCC 14579]	NP_832846.1	99.75/100	2125
*hlyII*	hemolysin II [Bacillus cereus ATCC 14579]	NP_833256.1	99.27/100	1738
*hlyR*	hemolysin II regulatory protein [Bacillus cereus]	AAO74585.1	95.02/100	1739
*inhA1*	immune inhibitor A precursor [Bacillus cereus ATCC 14579]	NP_831066.1	94.04/100	3987
*inhA2*	immune inhibitor A precursor [Bacillus cereus ATCC 14579]	NP_830479.1	98.87/100	4615
*bpsE*	Bacillus cereus exo-polysaccharide operon gene E [Bacillus cereus str. G9241]	EAL15982.1	79.79/95.59	198
*bpsF*	Bacillus cereus exo-polysaccharide operon gene F [Bacillus cereus str. G9241]	EAL15983.1	50.24/98.09	5558
*bpsH*	Bacillus cereus exo-polysaccharide operon gene H [Bacillus cereus str. G9241]	EAL15985.1	68.33/98.68	5576
*plcA*	1-phosphatidylinositol phosphodiesterase precursor [Bacillus cereus ATCC 14579]	NP_833485.1	95.71/99.09	1493
*^3^plcA*	1-phosphatidylinositol phosphodiesterase precursor [Bacillus cereus ATCC 14579]	NP_833485.1	55.35/99.39	147
*plcR*	transcriptional activator plcR	NP_835011.1	91.93/100	5486

^1^Nucleotide identity between NB125, NB176-1, and NB176 = 100%, ^2^ Amino acid sequence of *cerA* and *plcB* references are identical and were therefore reported together. ^3^located on plasmid 250-ppl.

Virulence gene typing revealed identical results for all strains tested (NB125, NB176-1, NB176) and were therefore reported only once. Amino acid (aa) identity and coverage percentages (id [%]/cov [%]) for each predicted virulence gene are given. To describe the genome position of all sequences detected by BTyper software, the corresponding reference aa sequences were downloaded and tblastn searched in Geneious Prime. Corresponding annotation numbers of NB125 are reported. Homologous genes (and their Prokka annotation numbers) for NB176-1 and NB176 can be found in the corresponding annotation table (**Supplementary Tables 1**, **8**).

Three out of nine bps genes belonging to the exo-polysaccharide operon *bpsX-H* were predicted. However, the sequence identities were relatively low (<70%) for two of these positive hits (*bpsF*, *bpsH*) and the corresponding CDS were located on different chromosomal loci. In general, predicted virulence genes were mostly located on the bacterial chromosome, except for *plcA*. This gene was not only predicted for the chromosome, but also for the 250-ppl with a lower nt sequence identity (**Table 9**). To verify this predicted plasmid-encoded phospholipase C gene despite the relatively low nt sequence identity, the corresponding aa sequence of ID_NB125/250-ppl_ = 147 was blastp searched in the NCBI database, revealing that the aa sequence is identical to a multispecies phosphatidylinositol-specific phospholipase C domain-containing protein (WP_086405655.1).

By using ABRicate and AMRFinderPlus software, the presence of antimicrobial resistance (AMR) genes was assessed *in silico*. For each of the three strains, the same AMR genes were predicted (**Table 10**) and the corresponding nucleotide sequences were identical. In total, 13 AMR genes were predicted for the bacterial chromosome (**Table 10**). These genes are associated with resistance to beta-lactam (*bla*, *bla2*), glycopeptide (*vanZ-F*), macrolide (*mhpL*, *abc-f*), lincosamide-streptogramin (*lsa*), streptogramin (*va*t), streptothricin (*sat A*), phenicol (*catA*) and fosfomycin (*fosB*) drug families. Three resistance genes, assigned to aminoglycoside class of antibiotics, were predicted for plasmid 250-ppl (**Table 10**). No resistance genes were predicted for plasmids 185-ppl, 137-ppl, 99-ppl, 68-ppl, 43-ppl, and 14-ppl.

**Table 10:** Antimicrobial resistance genes predicted for the three sequenced Btt strains.

Gene symbol	Class of antibiotics	Replicon	ID_NB125_	Accession no.	Algorithm	^1^Identity and coverage percentages (id [%]/cov [%])	Description
lsa	lincosamide/ streptogramin	chr	1740	WP_011133547.1	AMRFinderPlus (HMM)	81.71/100	Lsa family ABC-F type ribosomal protection protein
bla2	beta-lactam	chr	1843	WP_012261328.1	AMRFinderPlus (PARTIALP)	93.04/61.48	BcII family subclass B1 metallo-beta-lactamase
bla	beta-lactam	chr	2045	WP_003178584.1	AMRFinderPlus (HMM)	69.03/99.02	class A beta-lactamase
satA	streptothricin	chr	2077	A7J11_06109	ABRicate (db = NCBI)	91.67/98.38	streptothricin N-acetyltransferase SatA
vat	streptogramin	chr	2625	WP_063856935.1	AMRFinderPlus (HMM)	66.83/95.83	Vat family streptogramin A O-acetyltransferase
abc-f	macrolide	chr	2698	WP_003234144.1	AMRFinderPlus (HMM)	65.68/98.72	ABC-F type ribosomal protection protein
bla	beta-lactam	chr	2766	WP_063842248.1	AMRFinderPlus (BLASTP)	95.1/100	class A beta-lactamase Bla1
				AY453161:501-1430	ABRicate (db = ARG-ANNOT)	92.46/99.78	
				A7J11_01054	ABRicate (db = NCBI)	96.63/100	
				X06599:273-1194	ABRicate (db = CARD)	96.64/100	
bla	beta-lactam	chr	2815	WP_003178584.1	AMRFinderPlus (HMM)	65.68/96.74	class A beta-lactamase
				M15195	ABRicate (db = resfinder)	89.35/98.74	
catA	phenicol	chr	2933	WP_071218680.1	AMRFinderPlus (HMM)	57.87/99.54	type A chloramphenicol O-acetyltransferase
fosB	fosfomycin	chr	3252	WP_000943763.1	AMRFinderPlus (BLASTP)	99.28/100	FosB/FosD family fosfomycin resistance bacillithiol transferase
				A7J11_05167	ABRicate (db = NCBI)	99.04/100	
				NC_004722.1:1972252-1972669	ABRicate (db = CARD)		
				CP001903	ABRicate (db = resfinder)		
mphL	macrolide	chr	*3699+3700	A7J11_05208	ABRicate (db = NCBI)	92.65/99.89	macrolide 2’-phosphotransferase MphL
				ACMJ01000036:72075-72987	ABRicate (db = CARD)	92.66/99.89	
vanZ-F	glycopeptide	chr	4271	AF155139:4339-4959	ABRicate (db = ARG-ANNOT)	84.52/94.20	glycopeptides resistance protein VanZ-F
				A7J11_00543	ABRicate (db = NCBI)	84.52/94.20	
abc-f	macrolide	chr	5102	WP_063854496.1	AMRFinderPlus (HMM)	32.6/94.66	ABC-F type ribosomal protection protein
aadD1	aminoglycoside	250-ppl	43	WP_001014230.1	AMRFinderPlus (BLASTP)	96.84/100	aminoglycoside O-nucleotidyltransferase ANT(4’)-Ia
				AF181950:3176-3946	ABRicate (db = ARG-ANNOT)	95.20/100	
				A7J11_05311	ABRicate (db = NCBI)	95.20/100	
				NC_013342.1:26738-27500	ABRicate (db = CARD)	95.28/100	
				AF181950	ABRicate (db = resfinder)	95.20/100	
ant(4’)-I	aminoglycoside	250-ppl	54	WP_087346196.1	AMRFinderPlus (HMM)	82.35/86.33	ANT(4’)-I family aminoglycoside nucleotidyltransferase
ant(4’)-I	aminoglycoside	250-ppl	76	WP_001014230.1	AMRFinderPlus (HMM)	49.6/99.6	ANT(4’)-I family aminoglycoside nucleotidyltransferase

^1^Nucleotide identity between NB125, NB176-1 and NB176 = 100%. * Stop codon within sequence hit, therefore two CDS cover sequence hit.

ABRicate, including the ARG-ANNOT (2022-Jun-13, n = 1749), ResFinder (2022-Jun-13; n = 3144), CARD (2022-Jun-13; n = 2220), and NCBI AMRFinderPlus (2022-Jun-13; n = 6146) databases, was used with an 80% nucleotide identity and 70% coverage threshold. The tool AMRFinderPlus (database version 2022-05-26.1) was run in combined mode (nucleotide + protein) with default settings. Note that AMRFinderPlus uses curated BLAST and HMM cutoffs to optimize specificity and sensitivity of AMR detection. Therefore, results are reported, even if the identity and coverage values are below the default cutoff settings. If the same AMR gene was predicted using different algorithms and/or underlying databases, the results were combined into one row of the table. AMR gene typing revealed identical results for NB125, NB176-1, and NB176 and were therefore reported only once using the parental strain NB125 as reference. Homologous genes of NB176-1 and NB176 can be found in the corresponding annotation tables (**Supplementary Tables 1**, **8**).

### 3.6 *In silico* MLST, *panC* clade assignment and *rpoB* allelic typing

*In silico* analysis performed by BTyper software confirmed that the three strains NB125, NB176-1 and NB176 belonged to the *B. cereus* group. The different typing methods (*panC*, MLST, *rpoB*) revealed identical results for each Btt strain (**Table 11**). All three strains were assigned to *panC* clade 4 and the same closest-matching *B. cereus* group genome was reported. Analysis of seven MLST genes showed, that all three strains shared an identical allelic profile and were therefore assigned to the same sequence type (ST=23). Based on the *rpoB* sequence of each strain, the same allele type (AT0296) was identified, which showed a high similarity (100%) to *B. cereus s.l*.

**Table 11:** *In silico panC* clade typing, multi-locus sequence typing (MLST), and *rpoB* allelic typing of the three sequenced Btt strains.

*panC* ^1^ clade	Closest strain [id%/cov%]	ST^2^	MLST *Bacillus cereus* ^3^							*rpoB^4^ AT* [id%/cov%]
			*glp*	*gmk*	ilv	*pta*	*pur*	*pyc*	*tpi*	
4	B_cereus_172560W [98.47/100]	23	15	7	7	2	5	8	13	AT0296 [100/100]

^1^ pantoate-beta alanine ligase (ID_NB125_ = 3727), ^2^ sequence type, ^3^ glp: ID_NB125_ = 4210; gmk: ID_NB125_ = 1304; ilv: ID_NB125_ = 3480; pta: ID_NB125_ = 5451; pur: ID_NB125_ = 5003; pyc: ID_NB125_ = 1227, tpi: ID_NB125_ = 5717, ^4^ RNA polymerase β subunit (ID_NB125_ = 5220).

*In silico panC* clade typing, multi-locus sequence typing (MLST), and *rpoB* allelic typing was performed for each strain using BTyper software. The different typing methods revealed identical results for NB125, NB176-1, and NB176 and were therefore reported only once. The annotation IDs for NB125 are provided below the table. Homologous genes (and their IDs) for NB176-1, and NB176, can be found in the corresponding annotation table (**Supplementary Table 1**).

### 3.7 Phylogenetic analysis

Based on digital DNA-DNA hybridization (dDDH), twelve most closely related type strain genomes, all belonging to the *Bacillus cereus* group, were determined automatically using the TYGS server (**Supplementary Table 14** and **Supplementary Figure 1**). The most closely related type strain genome of NB125, NB176-1, and NB176 was *Bacillus thuringiensis* ATCC 10792 with a dDDH value of 68.3%, 68.1%, and 68.1%, respectively, followed by *Bacillus cereus* ATCC 14579 with a dDDH value of 65.6%, 65.6% and 65.7%, respectively (**Supplementary Table 14**). All pairwise dDDH values between the three genomes under assessment and the type strain genomes were below the species delineation threshold of 70%, but the upper limit of the 95% confidence interval regarding *Bacillus thuringiensis* ATCC 10792 was above 70%. According to dDDH, NB125, NB176-1, and NB176 are highly similar, with pairwise dDDH values ranging from 99.9 to 100% (**Supplementary Table 14**), resulting in the same species (70% dDDH species threshold) as well as subspecies (79% dDDH subspecies threshold) clustering (**Supplementary Table 14**, **Supplementary Figure 1**). The overall high sequence similarity of NB125, NB176-1 and NB176 was further confirmed by pairwise ANIb values ranging from 99.89 to 99.99% (**Supplementary Table 15**). Pairwise ANIb analysis of the three genome sequences and the closest type strains revealed that NB125, NB176-1 and NB176 showed ANIb values slightly above 95% with both *B. thuringiensis* and *B. cereus* type strain genomes (**Supplementary Table 15**).

## 4 Discussion

With the first and complete *de novo* reconstruction of the genome of NB125, the molecular changes that led to the creation of NB176-1 and NB176, the current production strain formulated in the plant protection product Novodor^®^ FC, could be traced. To decipher their complete genomes, a hybrid approach of short (Illumina) and long read (Nanopore) sequencing (Wick et al., 2017b) was applied, which allowed the detection of all genomic features underlining the strength of this technique. Long sequencing reads spanning repetitive regions helped to resolve these critical genomic features although manual intervention had been required to finalize the assembly (Wick et al., 2017a). The reliability of the genomic assemblies of NB125, NB176-1 and NB176 were supported by the recent complete genome assembly of *Bacillus thuringiensis* serovar *morrisoni* (strain 4AA1) (Genbank assembly GCA_022810725.1). Its chromosomal length, number and length of plasmids was similar to NB125, NB176-1 and NB176, although the applied sequencing technique was different (Illumina sequencing, PacBio (single molecule sequencing) and Sanger sequencing techniques). In general, resolving repetitive sequences and multi-copy genes compromising the correct assembly of high throughput sequencing data is key to a complete bacterial genome reconstruction. The challenge of unresolved plasmid sequences might be the reason why in a previous sequencing approach of NB176 (Biggel et al., 2021) the identification of the plasmid number and length was not successful.

In the literature, NB176-1 and NB176 were described as high-yielding derivatives of NB125 (Adams et al., 1994; Gurtler and Petersen, 1994; European Food Safety Authority, 2013). However, only little information was known on the differences between the parental strain NB125 and its radiation-derived strains. Although a duplication of the *cry3Aa* gene in NB176-1 was already predicted by Adams et al. (1994) by Southern hybridization and partial gene sequencing, a conclusive proof is finally provided by this study, identifying *cry3Aa* on plasmids 99-ppl and 185-ppl of NB176-1. Furthermore, the genomic analysis revealed that 99-ppl was a recombination product of the original 137-ppl plasmid and a region of 185-ppl carrying the *cry3Aa* gene and therefore resulting in its duplication and the Cry3Aa overproducing phenotype of NB176-1. By this finding, the origin of the *cry3Aa* gene could be unequivocally defined on the 185-ppl (~120 kDa) of NB125, which is close to the plasmid size of 90 kDa that had been proposed as location of *cry3Aa* (Sekar et al., 1987).

Besides the noted re-arrangement, we further propose that the shortening of 137-ppl, which led to the creation of 99-ppl, was mediated by Tn3 family transposases (TnBth1, TnBth2) and an IS4 family transposase IS231C. It was described previously that insertion sequences, such as IS231 and IS232 and transposons are frequently found in the vicinity of *cry* genes enabling transposition and gene duplication (Menou et al., 1990; Mahillon et al., 1994; Murawska et al., 2014; Fiedoruk et al., 2017). In addition to the *cry3Aa* gene, two other insecticidal genes, encoding for Mpp23Aa1 (old name: Cry23Aa) and Xpp37Aa1 (old name: Cry37Aa) were located on the original Cry3Aa encoding plasmid 185-ppl. These findings are in line with Caballero et al. (2020) who suggested that the commercial strain NB176 produces the three insecticidal proteins Cry3Aa, Mpp23Aa1 (Cry23Aa), and Xpp37Aa1 (Cry37Aa) (Caballero et al., 2020). These conclusions are difficult to draw from the previously published incomplete assembly of NB176 (Biggel et al., 2021) since collapsing repeat regions did not allow assumptions about changes and recombination in plasmid sequences. Genome assemblies are further challenged by the presence of repetitive mobile regions that could flank δ-endotoxin and antimicrobial resistance genes (Kronstad et al., 1983; Fiedoruk et al., 2017; Orlek et al., 2017) hiding their genomic position (He et al., 2015; Sheppard et al., 2016; Arredondo-Alonso et al., 2017). The exact location of antimicrobial resistance genes is crucial, because they might be spread when present on mobile genetic elements such as plasmids (Tschäpe, 1994; Thomas and Nielsen, 2005; Berbers et al., 2020).

Although NB176-1 and NB176 are almost identical in their plasmid sequences, a single chromosomal deletion of ~175.8 kbp was identified in NB176, when compared to NB125 and NB176-1. The deletion comprised a set of 182 CDS, of which 170 CDS had no additional copies on the chromosomal or any other plasmid sequences. Although sixty CDS located on the deletion site were used for functional analyses, it remains unclear how this deletion influence phenotypic characteristics. Some of the deleted genes might enable the parental strain NB125 to survive in its natural environment but may be unnecessary under production conditions. Since NB176-1 was deposited at the DSMZ in 1989 and NB176 is representing the current strain under commercial production, it can be hypothesized that the genome of NB176 in its current state is highly stable and did not undergo any further changes. Since the genomes of NB176-1 and NB176 are almost identical, the 175.8 kbp deletion could be regarded as a singular event in the history of NB176.

The screening for antimicrobial resistance genes and “*Bacillus cereus s.l.*” (Bc) virulence genes did not reveal differences between the parental and derivative strains. Regarding enterotoxin genes, all three Btt strains harbored genes encoding for the non-hemolytic enterotoxin (*nhe*) and hemolysin BL (*hbl*), but were negative for cytotoxin K (*cyt K*), consistent with previous studies concerning Btt (Kyei-Poku et al., 2007; Schwenk et al., 2020; Biggel et al., 2021; Bonis et al., 2021). Moreover, this study confirmed that the *nhe* promoter was disrupted by a transposase insertion in all three strains and thus that Nhe expression might be prevented in the commercial NB176 (Johler et al., 2018; Biggel et al., 2021). Genes encoding for one-component putative enterotoxins (*entFM*, *bceT*, *entA*) pore forming haemolysins (*clo*, *hlyII*), metalloproteases (*inhA1*, *inhA2*), phospholipases (*plcA*, *cerA*, *cerB*), and regulatory proteins (*hlyIIR*, *plcR*) were detected. It can be assumed that the three detected exo-polysaccharide genes (*bpsE/F/H*) are false positive hits, since only three out of nine *bpsX-H* genes were detected and located on different chromosomal loci. Previous studies have demonstrated that some members of the *B. cereus* group possess genes sharing a high degree of homology with Bps-encoding genes (Carroll et al., 2019; Carroll et al., 2020). Therefore, it was concluded that 19 chromosomally and one plasmid-encoded (putative phosphatidylinositol-specific phospholipase C located on plasmid 250-ppl) virulence genes were found.

Thirteen chromosomally encoded AMR genes were predicted. Four beta-lactamase genes were detected, in agreement with previous studies that found beta-lactamase genes as well as phenotypic resistance to be very common in the *B. cereus* group, including *B. thuringiensis* (Luna et al., 2007; Park et al., 2009; Kim et al., 2015; Yim et al., 2015; Fiedler et al., 2019; Bianco et al., 2021; Mills et al., 2022). Even though a single vancomycin resistance gene was found, a vancomycin resistance seems unlikely, as individual *van* genes were previously reported in the *B. cereus* group but were insufficient to confer phenotypic resistance (Arthur et al., 1992; Patel et al., 1997; Bianco et al., 2021; Mills et al., 2022). Genes associated with resistance to macrolide (*mhpL*, *abc-f*), lincosamide-streptogramin (*lsa*), streptogramin (*vat*), streptothricin (*sat A*), phenicol (*catA*) and fosfomycin (*fosB*) drug families were also detected in the three analyzed strains. In addition to the chromosomally encoded AMR genes, three nucleotidyltranferase genes associated with aminoglycoside resistance were predicted for plasmid 250-ppl. Some isolates of the *B. cereus* group are known for aminoglycoside resistance (Parulekar and Sonawane, 2018; Fiedler et al., 2019) whereas others carried nucleotidyltransferase genes but were not phenotypically resistant (Fiedler et al., 2019). Since only few studies associate the presence of AMR (Mills et al., 2022) or virulence genes to phenotypic traits, no direct conclusion can be drawn from *in silico* studies about the actual functionality of any of these genes or the conditions under which these genes might be expressed.

## Data availability statement

The datasets presented in this study can be found in online repositories. The names of the repository/repositories and accession number(s) can be found below: https://www.ncbi.nlm.nih.gov/, PRJNA886432.

## Author contributions

LS, RK, JJ, and JW designed the study. FV helped providing the Btt strain. LS conducted the experiments. LS performed the analysis of the sequences. LS wrote the original draft and RK, JJ and JW reviewed and edited the manuscript. All authors have read and agreed to the published version of the manuscript. All authors contributed to the article and approved the submitted version.
