# Supplementary material for: Elucidating the genomic history of commercially used Bacillus thuringiensis subsp. tenebrionis strain NB176
Source: Front Cell Infect Microbiol. 2023 Mar 20;13:1129177. doi: 10.3389/fcimb.2023.1129177 (PMC10067926; doi:10.3389/fcimb.2023.1129177)
Supplement: Supplementary file 1 [file DataSheet_1.docx]

Supplementary Material

# Supplementary Figures

**Supplementary Figure 1:** Tree inferred with FastME 2.1.6.1 (Lefort et al., 2015) from GBDP distances calculated from genome sequences. The branch length are scaled in terms of GBDP distance formula d_5_. The number above branches are GBDP pseudo-bootstrap support values >60% from 100 replications, with an average branch support of 86.6%. The tree was rooted at the midpoint (Farris, 1972). Leaf labels are annotated by affiliation to species (70% dDDH threshold) and subspecies (79% dDDH threshold) clusters, genomic G+C content (in %), δ values, total genome sequence length (in bp), number of proteins and an indication whether or not the strain was provided by the user. The three user-provided genomes NB125, NB176-1 and NB176 are shown in parentheses.

# Supplementary Tables

## Table captions for Excel files

**Supplementary Table 1:** Annotation table of the bacterial chromosome (chr). The annotations NB125 (DSM 5526), NB176-1 (DSM 5480) and NB176 were added by Prokka software (v1.14.6). Since all annotated features (CDS + RNA genes) were numbered consecutively by Prokka, the location and annotation of RNA genes were also included. Homology of RNA genes (highlighted in grey) was not investigated. CDS were compared for their homology using Roary software (v3.13). Annotation numbers of CDS that appear horizontally in one line have been identified as homologous. If an annotation (a homologous CDS) was missing, its absence is indicated by the letter m. The start, end and orientation of an annotation refer to the parental strain NB125.

**Supplementary Table 2:** Annotation table of the 14.9 kbp plasmid (14-ppl). Since nucleotide sequences of 14-ppl were identical between the three Btt strains NB125 (DSM 5526), NB176-1 (DSM 5480) and NB176 the plasmid was annotated only once. Automatic annotation was performed using Prokka software (v1.14.6) and annotations were numbered consecutively.

**Supplementary Table 3:** Annotation table of the 43.8 kbp plasmid (43-ppl). Since nucleotide sequences of plasmid 43-ppl were identical between the three Btt strains NB125 (DSM 5526), NB176-1 (DSM 5480) and NB176 the plasmid was annotated only once. Automatic annotation was performed using Prokka software (v1.14.6) and annotations were numbered consecutively.

**Supplementary Table 4:** Annotation table of the 68.5 kbp plasmid (68-ppl). Since nucleotide sequences of plasmid 68-ppl were identical between the three Btt strains NB125 (DSM 5526), NB176-1 (DSM 5480) and NB176, the plasmid was annotated only once. Automatic annotation was performed using Prokka software (v1.14.6) and annotations wre numbered consecutively.

**Supplementary Table 5:** Annotation table of the 99 kbp plasmid (99-ppl). This plasmid was found in both Btt strains NB176-1 (DSM 5480) and NB176. Since nucleotide sequences of 99-ppl were identical between the two strains, the plasmid was annotated only once. Automatic annotation was performed using Prokka software (v1.14.6) and annotations were numbered consecutively. Plasmid 99-ppl shared a large nucleotide region (highlighted in pink) with 137-ppl of the parental strain NB125 (DSM 5526) (Supplementary Table 6). Although there were some variations in the automatic prediction of CDS between the two plasmids, the corresponding nucleotide sequence was identical in this area. In addition to that shared region, a shorter part of 99-ppl (highlighted in blue) was also found in 185-ppl (Supplementary Table 7). This duplicated region is carrying the cry3Aa gene. ID_99ppl_ = 85 was additionally marked in blue, since this CDS is also present in 185-ppl.

**Supplementary Table 6:** Annotation table of the 137 kbp plasmid (137-ppl). This plasmid was only found in Btt strain NB125 (DSM 5526). Automatic annotation was performed using Prokka software (v1.14.6) and annotations were numbered consecutively. Plasmid 137-ppl shared a large nucleotide region (highlighted in pink) with 99-ppl of Btt strains NB176-1 (DSM 5480) and NB-176. Although there were some variations in the automatic prediction of CDS between the two plasmids, the corresponding nucleotide sequence was identical in this area. A total of 78 CDS (highlighted in red) were missing in both NB176-1 (DSM 5480) and NB-176 due to a large plasmid sequence deletion in this two strains.

**Supplementary Table 7:** Annotation table of the 185 kbp plasmid (185-ppl). The annotations of Btt strain NB125 (DSM 5526), NB176-1 (DSM 5480) and NB176 were added by Prokka software (v1.14.6) and were numbered consecutively. Protein coding DNA sequences (CDS) were compared for their homology using Roary software (v3.13). Annotation numbers that appear horizontally in one line have been identified as homologous. The start, end and orientation of an annotation refer to the parental strain NB125. Part of the 185-ppl (highlighted in blue) is also found in 99-ppl (Supplementary Table 5) of strain NB176-1 (DSM 5480) and NB176. This duplicated region is carrying the *cry3Aa* gene.

**Supplementary Table 8:** Annotation table of the 250.5 kbp plasmid (250-ppl). The annotations of Btt strains NB125 (DSM 5526), NB176-1 (DSM 5480) and NB176 were added by Prokka software (v1.14.6). Since all annotated features (CDS + RNA genes) were numbered consecutively by Prokka, the location and annotation of RNA genes were also included. Homology of RNA genes (highlighted in grey) was not investigated. CDS were compared for their homology using Roary software (v3.13). Annotation numbers of CDS that appear horizontally in one line have been identified as homologous. If an annotation (a homologous CDS) was missing, its absence is indicated by the letter m. The start, end and orientation of an annotation refer to the parental strain NB125.

**Supplementary Table 9:** Annotation table of the 182 CDS located on the chromosomal deletion in strain NB176. The annotations of Btt strains NB125 (DSM 5526), NB176-1 (DSM 5480) and NB176 were added by Prokka software (v1.14.6) and CDS were compared for their homology using Roary software (v3.13.). Annotation numbers of CDS that appear horizontally in one line have been identified as homologous. If an annotation (a homologous CDS) was missing, its absence is indicated by the letter m. The start, end, orientation, length, enzyme commission number (EC no.), and Cluster of Orthologous Groups number (COG no.) of an annotation refer to the parent strain NB125.

**Supplementary Table 10:** Reciprocal ANIb (ANI algorithm using BLAST) values [%] and aligned percentage (in parentheses) between the three genomes under assessment (NB125, NB176-1, and NB176) and the selected type-strain-genomes.

**Supplementary Table 11:** Closest type strain genomes of NB125, NB176-1, and NB176 based on digital DNA-DNA-Hybridization. The complete genome sequences of NB125, NB176-1 and NB176 were uploaded to the Type (Strain) Genome Server (TYGS) and closest type strains genomes were determined automatically.

## Supplementary Tables in Word format

**Supplementary Table 12:** Sequence identity matrix for all assembled plasmids. Plasmids were assembled *de novo* for each strain NB125 (DSM 5526), NB176-1 (DSM 5480) and NB176 from whole genome sequencing data. Sequence alignment was performed with Mauve progressive algorithm. Sequence identity [%] is the percentage of bases that are identical in the alignment.

| **Replicon** | **Length [bp]** |  | NB125 | NB176-1 |
| --- | --- | --- | --- | --- |
| **14-ppl** | 14,853 | NB176-1 | 100% |  |
|  |  | NB176 | 100% | 100% |
| **43-ppl** | 43,822 | NB176-1 | 100% |  |
|  |  | NB176 | 100% | 100% |
| **68-ppl** | 68,504 | NB176-1 | 100% |  |
|  |  | NB176 | 100% | 100% |
| **99-ppl** | 99,433 | NB176-1 | - |  |
|  |  | NB176 | - | 100% |
| **137-ppl** | 137,412 | NB176-1 | - |  |
|  |  | NB176 | - | - |
| **185-ppl** | 185,418 | NB176-1 | 100% |  |
|  |  | NB176 | 99.996% | 99.996% |
| **250-ppl** | 250,492 | NB176-1 | 99.966% |  |
|  |  | NB176 | 99.980% | 99.982% |
|  |  |  |  |  |

**Supplementary Table 13:** Nucleotide sequence identity matrix for the bacterial chromosome of NB125, NB176-1, and NB176. Multiple sequence alignment (MSA) was performed with Mauve progressive algorithm, followed by manual left-realignment. Sequence identity [%] is the percentage of bases that are identical in the alignment. Nucleotide identity after excluding the chromosomal deletion (178,499 bp) of NB176 is given in parentheses.

| **Chromosome** | NB125 | NB176-1 |
| --- | --- | --- |
| NB176-1 | 99.955% [99.954%] |  |
| NB176 | 96.770% [99.952%] | 96.798% [99.981%] |

**Supplementary Table 14:** Pairwise digital DNA-DNA-Hybridization (dDDH) values between NB125 (DSM 5526), NB176-1 (DSM 5480), and NB176 and the selected type-strain-genomes. The dDDH values [%] and 95% confidence intervals [%] were calculated using the recommended formula d_4_. Table is ordered by dDDH value in descending order. Assembly accession numbers of type strain genomes are available in the Supplementary material (Supplementary Table 11).

|  | **NB125**  (DSM 5526) | **NB176-1**  (DSM 5480) | **NB176** |
| --- | --- | --- | --- |
| NB176-1 (DSM 5480) | 100 [100 - 100] |  |  |
| NB176 | 100 [99.9 - 100] | 99.9 [99.9 - 100] |  |
| *Bacillus thuringiensis* ATCC 10792 | 68.3 [65.3 - 71.1] | 68.1 [65.1 - 70.9] | 68.1 [65.2 - 71.0] |
| Bacillus cereus ATCC 14579 | 65.6 [62.7 - 68.5] | 65.6 [62.7 - 68.5] | 65.7 [62.8 - 68.6] |
| *Bacillus toyonensis* NCIMB 14858 | 45.4 [42.8 - 48.0] | 45.4 [42.9 - 48.0] | 45.4 [42.8 - 48.0] |
| *Bacillus tropicus* N24 | 44.9 [42.4 - 47.5] | 44.9 [42.4 - 47.5] | 45.0 [42.5 - 47.6] |
| *Bacillus fungorum* 17-SMS-01 | 44.7 [42.2 - 47.3] | 44.7 [42.2 - 47.3] | 44.9 [42.3 - 47.4] |
| *Bacillus wiedmannii* FSL W8-0169 | 44.5 [41.9 - 47.1] | 44.5 [41.9 - 47.1] | 44.5 [42.0 - 47.1] |
| *Bacillus paranthracis* MCCC 1A00395 | 44.2 [41.6 - 46.7] | 44.1 [41.6 - 46.7] | 44.2 [41.6 - 46.7] |
| *Bacillus anthracis* ATCC 14578 | 44.1 [41.6 - 46.7] | 44.1 [41.6 - 46.7] | 44.2 [41.6 - 46.7] |
| *Bacillus albus* N35-10-2 | 43.9 [41.4 - 46.4] | 43.9 [41.4 - 46.4] | 43.8 [41.3 - 46.4] |
| *Bacillus luti* MCCC 1A00359 | 43.4 [40.9 - 46.0] | 43.4 [40.9 - 46.0] | 43.5 [41.0 - 46.1] |
| *Bacillus mobilis* MCCC 1A05942 | 43.2 [40.7 - 45.8] | 43.2 [40.7 - 45.8] | 43.3 [40.8 - 45.9] |
| *Bacillus proteolyticus* MCCC 1A00365 | 39.6 [37.1 - 42.1] | 39.6 [37.1 - 42.1] | 39.6 [37.1 - 42.1] |

**Supplementary Table 15:** Pairwise ANIb (ANI algorithm using BLAST) values between the three genomes under assessment and the selected type-strain-genomes. Reported ANIb values is the average value of two reciprocal values. Table is ordered by ANIb values of the parental strain NB125 in descending order. Assembly accession numbers of type strain genomes, reciprocal values of all pairwise comparisons (all vs. all) as well as aligned percentages are available in the supplementary material (Supplementary Table 11, 10).

|  | **NB125**  (DSM 5526) | **NB176-1**  (DSM 5480) | **NB176** |
| --- | --- | --- | --- |
| NB176-1 (DSM 5480) | 99.99 |  |  |
| NB176 | 99.89 | 99.90 |  |
| *Bacillus thuringiensis* ATCC 10792 | 95.37 | 95.31 | 95.24 |
| *Bacillus cereus* ATCC 14579 | 95.33 | 95.31 | 95.23 |
| *Bacillus toyonensis* NCIMB 14858 | 91.09 | 91.09 | 91.04 |
| *Bacillus tropicus* N24 | 90.97 | 90.95 | 90.93 |
| *Bacillus wiedmannii* FSL W8-0169 | 90.93 | 90.91 | 90.86 |
| *Bacillus paranthracis* MCCC 1A00395 | 90.69 | 90.66 | 90.59 |
| *Bacillus albus* N35-10-2 | 90.67 | 90.67 | 90.66 |
| *Bacillus anthracis* ATCC 14578 | 90.64 | 90.63 | 90.61 |
| *Bacillus fungorum* 17-SMS-01 | 90.62 | 90.63 | 90.59 |
| *Bacillus luti* MCCC 1A00359 | 90.51 | 90.48 | 90.47 |
| *Bacillus mobilis* MCCC 1A05942 | 90.41 | 90.42 | 90.41 |
| Bacillus *proteolyticus* MCCC 1A00365 | 88.94 | 88.91 | 88.87 |
