# Supplementary figures and images for: Elucidating the genomic history of commercially used Bacillus thuringiensis subsp. tenebrionis strain NB176
Source: Front Cell Infect Microbiol. 2023 Mar 20;13:1129177. doi: 10.3389/fcimb.2023.1129177 (PMC10067926; doi:10.3389/fcimb.2023.1129177)

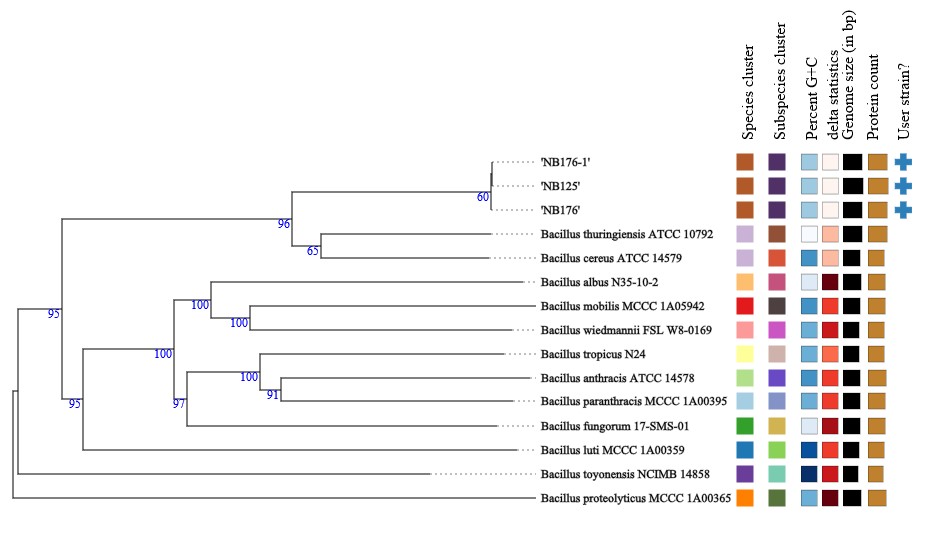

Supplement: Supplementary file 2 [file Image_1.jpeg]
